# Ciliated Hepatic Foregut Cyst: A Report of a Case Incidentally Discovered during Transplant Evaluation

**DOI:** 10.1155/2019/7828427

**Published:** 2019-10-09

**Authors:** Thomas Enke, Wuttiporn Manatsathit, Shaheed Merani, Kurt Fisher

**Affiliations:** ^1^The University of Nebraska Medical Center, NE, USA; ^2^Division of Gastroenterology and Hepatology, University of Nebraska Medical Center, Omaha, NE, USA; ^3^Department of Transplant Surgery, University of Nebraska Medical Center, Omaha, NE, USA; ^4^Department of Pathology, University of Nebraska Medical Center, Omaha, NE, USA

## Abstract

Ciliated hepatic foregut cyst (CHFC) is a rare benign cyst of the liver derived from an embryonic remnant of foregut epithelium. CHFC is typically asymptomatic and is found incidentally. Recent reports of malignant transformation may warrant surgical removal of CHFC. We present the case of a 54-year-old male who was discovered to have a CHFC while undergoing kidney transplant evaluation.

## 1. Introduction

Ciliated hepatic foregut cyst (CHFC) is a rare benign cyst of the liver derived from an embryonic remnant of foregut epithelium [1]. Patients with CHFC are almost always asymptomatic and mostly incidentally found on abdominal imaging. Although CHFC is benign and generally appears as a simple cyst on imaging, it has been reported to mimic appearance of hepatic cystic neoplasm on cross sectional imaging and rarely reported to be associated with malignancy [2]. With increasing utilization of abdominal cross images, and advancement in computerized tomography (CT) and magnetic resonance imaging (MRI), incidental hepatic cysts are increasingly detected, yet CHFC remains extremely uncommon [3]. Herein, we present a case of CHFC resembling a hepatic cystic neoplasm detected during kidney transplant evaluation.

## 2. Case Report

**Figure 1:**
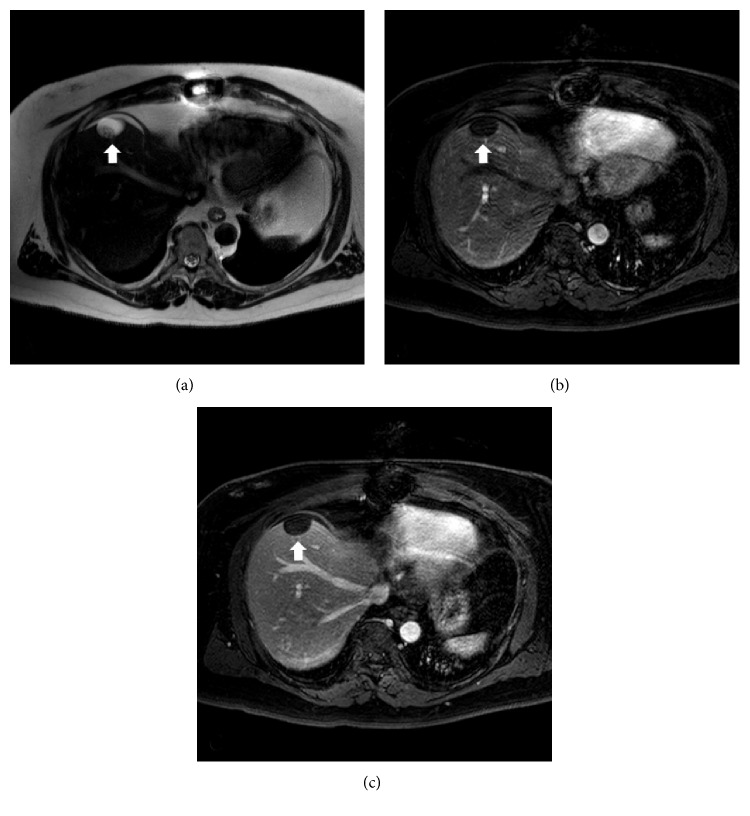
Transverse abdominal MRI of hepatic cyst. (a) T2-weighted image. (b) Arterial phase following intravenous contrast. (c) Venous phase following intravenous contrast.

A 54-year-old male with end stage renal disease undergoing kidney transplant evaluation presented to hepatology clinic for further evaluation of a hepatic cyst. The cyst was incidentally found on abdominal CT, and the patient was completely asymptomatic. A noncontrast abdominal CT scan revealed a 2.3 cm lesion of low attenuation in segment IVa of the liver. Additional workup including, bilirubin, ALT, AST, GGT, hepatitis B, C, and HIV screening were unremarkable. A subsequent contrast enhanced abdominal magnetic resonance imaging (MRI) scan confirmed a 2.8 cm hypodense cyst with a 1.7 cm solid component in the posterior aspect of the cyst with enhancement. A follow-up contrast enhanced MRI was performed, which demonstrated persistent of solid component (Figure 1).

**Figure 2:**
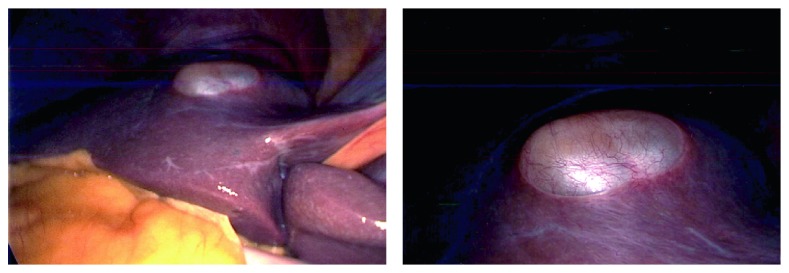
Intra-operative view of ciliated hepatic foregut cyst.

**Figure 3:**
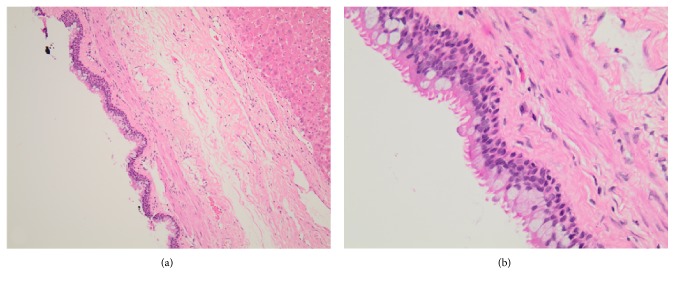
Histological sample of ciliated hepatic foregut cyst with hematoxylin and eosin staining. (a) Low power magnification. (b) High-power magnification.

Considering the persistent 1.7 cm solid component in the cyst, concern of hepatic cystic neoplasm was entertained. Additionally, immunosuppression after kidney transplant may facilitate progression of a neoplastic process if left untreated. Therefore, the decision was made to proceed with surgical resection. A laparoscopic approach was initially performed, which revealed a superficial hepatic cyst within segment IV of the liver, consistent with prior imaging. Given the location of the cyst, it was decided to convert to an open surgical approach. A tan, thin-walled, mucin-filled cyst (2.7 × 2.0 × 1.1 cm) was removed via cyst enucleation without complication (Figure 2). The patient received hemodialysis post-operative day (POD) 1, was advanced to a general diet POD 2, and was discharged home on POD 3. Histology revealed a ciliated pseudostratified epithelium consistent with a CHFC (Figure 3).

## 3. Discussion

CHFC is a rare benign hepatic cyst originally described by Wheeler and Edmonson as a cystic remnant of foregut epithelium similar to bronchogenic and esophageal cysts [1]. A literature review discovered 109 reported cases since 1964. Histologically, CHFCs classically consist of 4 layers, including a ciliated pseudostratified columnar epithelium, subepithelial connective tissue, a smooth muscle layer, and an outer fibrous capsule [1]. CHFCs are typically discovered during the fifth decade of life, but may present at any age [2–4]. Most patients are asymptomatic at time of diagnosis. When symptomatic, the majority present with right upper quadrant abdominal pain [2, 3]. However, a few cases of CHFCs causing jaundice from biliary obstruction have been reported [5, 6]. Classically, CHFCs are located in the left lobe of the liver, specifically segment IV, but can present within the right lobe as well [2, 3]. Additionally, extrahepatic locations, such as the gallbladder and extrahepatic biliary system, have been reported [2, 6–8].

Radiographic imaging is usually insufficient for diagnosis given the variability in appearance. Typically, CHFCs are small (<4 cm), subcapsular, unilocular, and fluid-filled, but may present with findings suggestive of solid debris as seen in the above patient [2, 3, 10, 11]. Ultrasound evaluation usually reveals a hypoechoic cyst and hyperdense without contrast enhancement on CT imaging [10]. Large variability is seen on MRI T1-weighted imaging, but CHFCs are nearly exclusively hyperintense on T2-weighted imaging [3, 10, 11]. The variety of imaging findings provide for a large differential which includes, simple cysts, mucinous cystic neoplasm (MCN), pyogenic abscess, amebic abscess, hydatid cyst, intrahepatic pseudocyst, biliary cystadenoma, and cystadenocarcinoma [12].

Definitive diagnosis is made through histology which reveals a pseudostratified columnar epithelium, subepithelial connective tissue layer, smooth muscle layer, and outer fibrous capsule [1]. Presence of cartilage and respiratory glands would be suggestive of a bronchogenic cyst and are absent in CHFCs. Fine needle aspiration (FNA) cytology may provide for a nonsurgical diagnosis with reports of a positive predictive value of 76% [3, 13]. The finding of pseudostratified squamous epithelium in a mucoid background is nearly diagnostic, given the absence of other liver pathology with similar cytology [13].

Although CHFCs were originally believed to be benign, more recent reports of CHFCs undergoing malignant transformation have challenged that dogma [14, 16]. Rates of malignant transformation were reported to range from 3% to 5% with squamous cell carcinoma being the most common malignancy [2, 3]. Size appears to be the largest risk factor for malignant transformation in CHFCs [2, 3]. Rates of malignant transformation are lower than those seen in MCN, which may be as high as 10% [17]. The role of carbohydrate antigen (CA) 19-9, and/or carcinoembryonic antigen (CEA) as a marker of malignant progression in CHFC does not appear helpful. There have been reports of patients with CHFC discovered to have elevated serum CEA, and elevated intra-cystic CEA and CA19-19 in the absence of malignancy [6, 16]. The current management of CHFC remains controversial. Given the potential risk of malignant transformation, most agree that surgical excision, increasingly performed through a laparoscopic approach, should be performed if the cyst is greater than 4–5 cm, symptomatic, growing, or possesses wall abnormalities on imaging [18, 19]. In the absence of radiographic features concerning for malignancy, a more conservative approach with serial imaging could be considered given the overall low risk of malignant transformation, particularly when lacking the above features.

CHFC is a rare cyst, but has been more frequently diagnosed in recent years. Given the potential for malignant transformation, it is an important diagnosis to consider in the setting of a hepatic cyst. Due to the limited number of cases, further characterization and understanding of the disease is important.
